# From gene discovery to new biological mechanisms: heparanases and congenital urinary bladder disease

**DOI:** 10.1093/ndt/gfv309

**Published:** 2015-08-27

**Authors:** Neil A. Roberts, Emma N. Hilton, Adrian S. Woolf

**Affiliations:** 1 Institute of Human Development, Faculty of Medical and Human Sciences, University of Manchester, Manchester Academic Health Science Centre, Manchester, UK; 2 Royal Manchester Children's Hospital, Manchester, UK

**Keywords:** growth, HPSE, LRIG, neuron, receptor, signalling

## Abstract

We present a scientific investigation into the pathogenesis of a urinary bladder disease. The disease in question is called urofacial syndrome (UFS), a congenital condition inherited in an autosomal recessive manner. UFS features incomplete urinary bladder emptying and vesicoureteric reflux, with a high risk of recurrent urosepsis and end-stage renal disease. The story starts from a human genomic perspective, then proceeds through experiments that seek to determine the roles of the implicated molecules in embryonic frogs and newborn mice. A future aim would be to use such biological knowledge to intelligently choose novel therapies for UFS. We focus on heparanase proteins and the peripheral nervous system, molecules and tissues that appear to be key players in the pathogenesis of UFS and therefore must also be critical for functional differentiation of healthy bladders. These considerations allow the envisioning of novel biological treatments, although the potential difficulties of targeting the developing bladder *in vivo* should not be underestimated.

## INTRODUCTION: GENETIC INSIGHTS INTO KIDNEY MALFORMATIONS

Renal tract malformations (RTMs) are primary diagnoses in ∼50% of children with end-stage renal disease (ESRD) [1]. RTMs can also cause incident ESRD throughout adult life [2]. In fact, it has been reasoned that RTMs are underreported in young adults with ESRD and that RTMs are likely to be primary diagnoses in a major subset of these individuals [3]. Mutations of genes that are normally expressed in the developing metanephros, or embryonic kidney, have been identified in some people with malformed kidneys [4–6]. These discoveries are impacting on personalized medicine pathways by refining diagnoses, clarifying requirements for long-term follow-up, and facilitating more informed genetic counselling [7, 8].

Genetic advances can also prompt studies to define the aberrations of cell and developmental biology that generate kidney malformations. In turn, such knowledge should facilitate the design of novel therapies for these disorders, to date generally considered to be intractable anatomical anomalies. These ideas are illustrated by the following example. Human mutations of *Fraser syndrome 1* (*FRAS1*), encoding a matrix molecule coating the outer surface of developing kidney tubules, causes bilateral renal agenesis (i.e. both kidneys and ureters are absent) [9]. Modelling this disease in *Fras1* mutant mice demonstrated impaired growth factor signalling in cells normally destined to form the rudimentary kidney and ureter [10, 11]. Strikingly, renal agenesis could be avoided by treatment with fibroblast growth factor 10 (FGF10) or glial cell line-derived growth factor (GDNF), which restore levels of phospho-extracellular signal-regulated kinase (pERK) [10, 11]. This molecule is part of an intracellular signalling pathway driving ureteric bud elongation to form the ureter stalk and bud branching to form kidney collecting ducts [12]. FGF10 and GDNF cell-surface receptors are receptor tyrosine kinases (RTKs), and we will allude to this class of molecules, as well as to pERK, when discussing the cell biology of a type of inherited bladder disease.

## CONGENITAL BLADDER DISEASES AND UROFACIAL SYNDROME

Congenital urinary bladder malformations include persistent cloaca (failure of the forming the bladder to separate from the hindgut), exstrophy (failure of ventral closure of the bladder), and bladder outlet obstruction (BOO), which itself can have anatomical (e.g. urethral valves) or functional causes (discussed below). Therapies for these malformations generally comprise prenatal and post-natal surgery to refashion and/or deobstruct structurally abnormal urinary tracts [13]. Such interventions are inevitably undertaken after bladder disease is well established, but they are the patients' only therapeutic options in view of our overwhelming ignorance of the primary causes of these anomalies. As reviewed [14], compared with our substantial knowledge of mutations that cause human kidney malformations, the genetic bases of bladder malformations are only just beginning to be defined. By analogy with the *FRAS1* kidney story, genetic insights into bladder disease might allow us to understand the biological pathogeneses of congenital bladder anomalies and conceive of novel treatments.

In this review we will focus on one such disease, urofacial syndrome (UFS), which has also been called Ochoa syndrome, after the surgeon who first described it. UFS is an autosomal recessive disorder featuring functional BOO and, although uncommon, it can be fatal, with a high incidence of associated ESRD in historical series [15]. We recently reviewed UFS's clinical features and disease-causing mutations [16, 17], so will only mention these aspects briefly here. Instead, we will focus on emerging ideas about the cell biology of UFS, prompted by genetic discoveries. In doing so, we will discuss heparanase proteins and the peripheral nervous system, molecules and tissues that appear to be key players in the pathogenesis of UFS and so which must also, by implication, be critical for functional differentiation of healthy bladders.

## UFS BLADDERS FAIL TO UNDERGO FUNCTIONAL DIFFERENTIATION

The human bladder rudiment has separated from the hindgut by 7 weeks of gestation [6, 18]. From this timepoint, detrusor smooth muscle (DSM) begins to differentiate from mesenchymal cells surrounding the endoderm-derived differentiating urothelium [18]. Based on mouse experiments, urinary tract SM differentiation is driven by sonic hedgehog (SHH), a urothelial-derived growth factor that initiates a molecular cascade in adjacent mesenchymal cells, causing them to upregulate cytoskeletal proteins mediating muscle contraction [19, 20]. Human lower urinary tract malformations have been associated with mutations in genes coding for SM (i.e. smooth muscle actin γ2 and smooth muscle heavy chain 11) and urothelial (i.e. uroplakin 3A) structural proteins [21–23].

**FIGURE 1:**
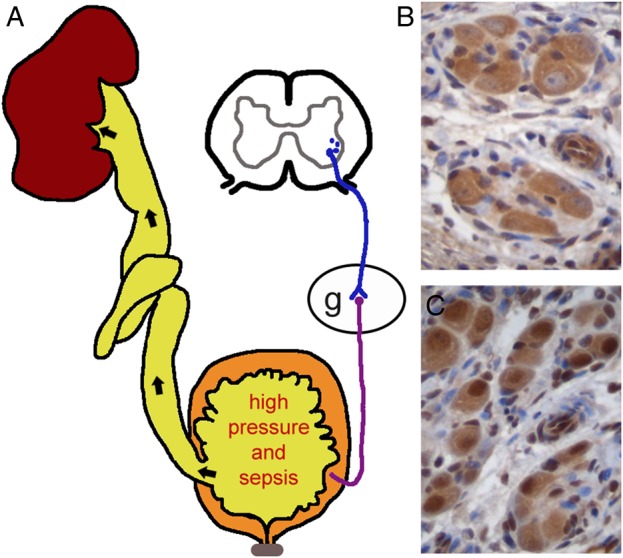
UFS clinical disease and implicated molecules. (**A**) The cartoon depicts urinary tract pathology in UFS. Note that, for simplicity, only one renal unit and neural circuit are shown. Note the dyssynergic, high pressure bladder, in which the detrusor contracts against a non-relaxed sphincter. Instead of efficiently exiting *per* urethra, urine stagnates in the bladder, with a high risk of urosepsis. High pressure vesicoureteric reflux of infected urine (black arrows in the ureter) causes recurrent pyelonephritis and parenchymal scarring with the risk of ESRD. On the right side of the cartoon, the autonomic innervation of the bladder is shown. A preganglionic neuron (blue) originates in the spinal cord and synapses within a ganglion (g) with a postganglionic neuron (purple). The latter innervates the bladder detrusor muscle (orange). This general scheme is similar for parasympathetic and sympathetic neurons, although the latter also innervate the internal sphincter. (**B** and **C**) Immunohistochemistry of a wild-type mouse pelvic ganglion showing HPSE2 (brown colour in B) and LRIG2 (brown colour in C) in neuronal cell bodies. One hypothesis is that, if either protein is absent, then the differentiation and/or function of parasympathetic and/or sympathetic nerves is perturbed and that this leads to functional BOO. Another, not mutually exclusive, idea is that the function of the external sphincter, skeletal muscle supplied by somatic motor nerves, is dysfunctional in UFS.

In UFS, the major anatomical steps of bladder development appear to be normal. In other words, the bladder has separated from the hindgut and contains DSM. Instead, UFS bladders fail to become fully differentiated in a physiological sense. The normal mature bladder acts as a low-pressure urinary reservoir that intermittently and completely expels its contents via the urethra [24]. In contrast, the UFS bladder fails to empty completely; this is an example of functional BOO since there is no anatomical obstruction within the urethral lumen [15, 25–27]. Cystometry in children with UFS typically reveals that the DSM contracts at the same time as the bladder sphincter [15, 25–27]. This dyssynergy results in urine pooling in the bladder lumen, with a consequent high risk of urosepsis. Moreover, because this urine is under high hydrostatic pressure, vesicoureteric reflux often occurs, with the risk of recurrent bacterial pyelonephritis, kidney parenchymal scarring, systemic hypertension and ESRD [15, 25–27]. This sequence of events is depicted in Figure 1A.

Healthy human bladders also undergo cyclical filling and voiding before birth [28]. Furthermore, ligating the urethra in foetal sheep leads to persistently and markedly raised intravesical pressures [29]. Foetal ultrasonographic anomaly screening of individuals who are later diagnosed as having UFS can show megacystis, or a grossly dilated bladder, and/or dilated ureters [25, 27]. These appearances suggest that functional BOO and raised intravesical pressures must occur from the prenatal period in UFS. Thus the bladder defect in UFS is clearly a developmental disorder.

## PERIPHERAL NEUROPATHIES MAY EXPLAIN THE CLINICAL FEATURES OF UFS

In the healthy bladder, voiding is driven by DSM contraction mediated by signalling through the parasympathetic arm of the autonomic nervous system [24] (Figure 1A). Indeed, biallelic mutation of *CHRM3* causes a human syndrome featuring congenital megacystis and hypocontractile bladders [30]. *CHRM3* codes for a muscarinic receptor, called M3, that is expressed by DSM cells and binds acetylcholine released by postganglionic parasympathetic neurons. Sympathetic noradrenergic signalling mediates both detrusor relaxation and internal sphincter closure [24]. Postnatally, higher central nervous system (CNS) centres modulate micturition, and voiding can be voluntarily impeded by external sphincter contraction mediated by somatic motor nerves [24].

As well as having functional BOO, people with UFS also have a characteristic grimace when smiling, laughing and crying [15, 16]. On occasion, more extensive skeletal muscle weakness has been described [27]. A neurogenic basis (or bases) for the bladder and facial defects in UFS has long been postulated [15]. Although there has been speculation about the anatomical site (or sites) of the neuropathology [15, 31], it is clear that people with UFS have no gross anatomic lesions, such as spina bifida. Our contention is that UFS's pathobiology includes both a somatic motor neuropathy affecting the VIIth cranial nerve, which innervates facial skeletal muscles, and an autonomic motor neuropathy affecting nerves supplying the urinary bladder. This working model is based on recent biological insights that followed the discoveries of genes mutated in UFS.

## HEPARANASE MOLECULES AND UFS

In 2010, our local research group [25] and a USA group [32] reported biallelic *HPSE2* mutations in a subset of families with UFS. *HPSE2* codes for HPSE2 (also called heparanase 2 or HPA2), a secreted protein with 40% homology to HPSE1 (also called heparanase 1 or HPA1) [33, 34]. HPSE1's biochemical actions have been intensively studied and are summarized here as a prelude to discussing HPSE2 in UFS.

Heparan sulphate proteoglycans (HPSGs) include the syndecans, inserted into plasma membranes, glypicans, linked to plasma membranes via anchors, and perlecan, collagen VIII and agrin, located in the extracellular matrix [35]. HPSE1 has endo-β-glucuronidase activity that degrades the HS side-chains of HSPGs [34, 36]. Various growth factors bind to HS side-chains [35] and HPSE1's heparanase enzymatic activity (hereafter abbreviated to HEA) releases them from core PGs. This model has been most studied regarding FGF functioning, for example, in angiogenesis [37] and branching morphogenesis [38]. HSPGs can also bind other growth factors (e.g. GDNF, SHH and bone morphogenetic proteins), so availabilities of these molecules may also be modulated by HEA. After HEA-mediated release from HSPG cores, growth factors remain attached to HS fragments, which themselves enhance binding of the growth factors to their cell-surface receptors; in the case of FGFs, these are RTKs [39, 40]. Growth factor binding triggers RTK phosphorylation, the first step in intracellular signalling cascades modulating growth and differentiation. HEA also enhances secretion of exosomes rich in growth factors and PGs, and this may also impact on signalling [41].

While HPSE1 is generally a cytoplasmic or a cell-surface associated protein, it has also been detected in cell nuclei [42], where it may modify gene transcription. HPSE1 has activities independent of HEA, and these include enhancing nerve growth factor–mediated neuritogenesis [43] and modulating cell adhesion and spreading [44]. Thus HPSE1 is a multifunctional protein and has been implicated in mediating metastasis, inflammation and certain complications of diabetes mellitus [45, 46].

## *HPSE2* AND *LRIG2* MUTATIONS CAUSE UFS

HPSE2 was cloned in 2000 [33], yet over the next decade little was known about its functions or biological roles. In 2010, Levy Adam *et al*. [34] reported that, unlike HPSE1, HPSE2 has no HEA; instead, by binding HPSE1 and also sequestering HSPG targets, HPSE2 inhibits HPSE1's HEA. In the same year, as mentioned above, *HPSE2* mutations were first reported in UFS [25, 32]. A more recent study [27] described a series including seven UFS families with *HPSE2* mutations; upon reviewing these and the previous cases, it was noted that the implicated *HPSE2* variants were often frameshift or stop mutations (i.e. there would be no functional HPSE2 protein made).

The genetic story became more complex when Stuart *et al.* [26] reported that some UFS patients lacking *HPSE2* mutations have biallelic mutations of *LRIG2*, encoding leucine-rich repeats and immunoglobulin-like domains 2. LRIG2 belongs to a family of three single-pass transmembrane proteins [47]. Most is known about LRIG1, which is a tumour suppressor, downregulating growth factor signalling by ubiquitination-mediated RTK degradation and inhibition of RTK recruitment to lipid rafts. The latter mechanism underlies LRIG1's ability to block GDNF-induced neuritogenesis *in vitro* [48].

**FIGURE 2:**
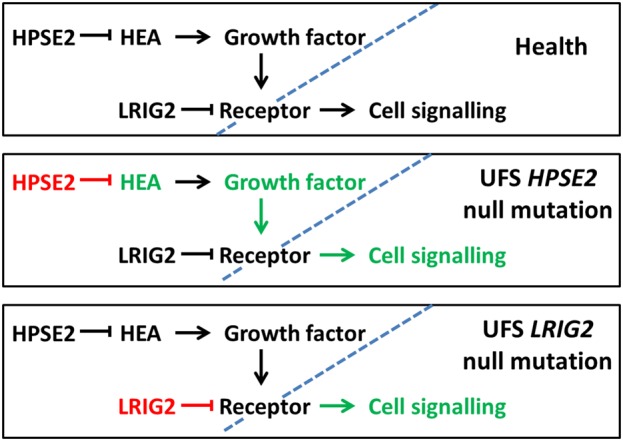
Potential aberrations in cell signalling in UFS differentiating tissues. In each of the three frames, the blue dotted line represents the border between the extracellular milieu (on the left) and the interior of the cell. *Top frame*: In health, HPSE2 serves to limit heparanase enzymatic activity (HEA) which releases growth factors from heparanase sulphate proteoglycans. The growth factors bind to cell-surface receptors and elicit intracellular signalling which controls growth and differentiation. LRIG2 serves as an independent check of receptor activity. *Middle frame*: When there are biallelic null mutations of *HPSE2*, there is no HPSE2 (red) and HEA is unchecked, leading to increased growth factor availability (green) and upregulated, abnormal, cell signalling (green). *Bottom frame*: When there are biallelic null mutations of *LRIG2* (red), there is no LRIG2 to check receptor-mediated cell signalling (green). The biological effect on cell signalling of loss of either HPSE2 or LRIG2 proteins are identical because they each regulate the same receptor(s). This would explain the observation that null mutations of *HPSE2* or *LRIG2* cause identical UFS phenotypes.

Little is known about LRIG2, although it has been shown to be permissive for glial tumour growth *in vivo* [49], and in a glioma cell culture model, LRIG2 interacts with epidermal growth factor receptor and modulates intracellular signalling [50]. UFS phenotypes of *HPSE2* or *LRIG2* mutation patients appear identical, so HPSE2 and LRIG2 probably work in related pathways. One hypothesized model, in which lack of HPSE2 or LRIG2 has the same detrimental outcome on cell signalling, is depicted in Figure 2.

## IMPLICATING UFS PROTEINS IN THE PERIPHERAL NERVOUS SYSTEM

In normal embryonic mice, HPSE2 and LRIG2 can be immunodetected in nerves growing into facial mesenchyme that will form skeletal muscles [27]. In healthy human embryos, both HPSE2 and LRIG2 proteins are immunodetected in peripheral, presumed autonomic, motor nerves growing into forming bladders [26]. In developing mice, similar results were found and both proteins were also detected in neural cell bodies in pelvic (parasympathetic) (Figure 1B and C) and lumbar (sympathetic) autonomic ganglia [27]. HPSE1 is also immunodetected in these ganglia [27], so HPSE2 would be well-placed to block HEA within the peripheral nervous system (Figure 2, upper frame).

These emerging data place HPSE2 and LRIG2 within developing peripheral nerves. Growth factors, including several bound by HSPGs, are implicated in mediating key steps of motor neuron differentiation, including the specification of neuron precursor cells, axonal growth from these cells and synaptogenesis at neuromuscular junctions [48, 51, 52]. What is less well understood, however, is how these processes are regulated so that growth factor signalling is tuned to avoid over- or underactivity, either of which could fatally compromise the generation of functional neuromuscular units. Our hypothesis is that HPSE2 and LRIG2 constitute key components of such a regulatory network that, when malfunctioning, causes UFS.

## MODELLING UFS SOMATIC MOTOR NEUROPATHY IN *XENOPUS*

**FIGURE 3:**
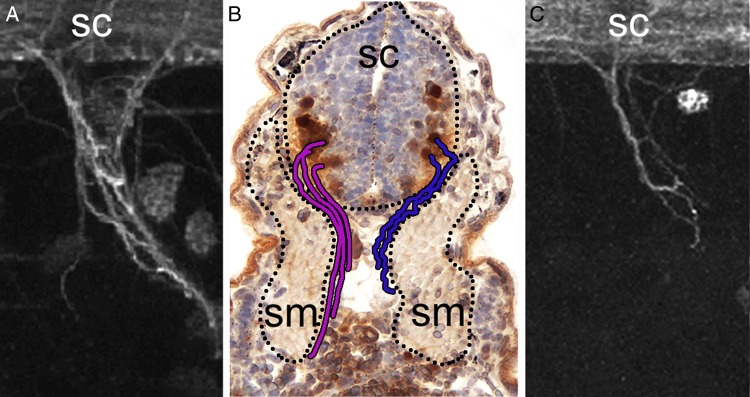
HPSE2 is required for peripheral nerve development in embryonic frogs. (**A**) Side view of a motor nerve which is growing out from the embryonic sc. In this whole mount preparation, neurons were immunostained (white) for acetylated α-tubulin. (**B**) Histology of transverse section of a tadpole trunk, with the animal's back at the top of the frame. The paths of two motor nerves have been sketched onto the photomicrograph. Their axons originate in cell bodies (immunostained for pERK in brown) located in ventrolateral domains of the spinal cord (sc), and grow towards blocks of developing skeletal muscle (sm). The normal nerve course is shown on the left (in purple), whereas a dysmorphic nerve is shown on the right (in blue). (**C**) Side view of a dysmorphic motor nerve which is growing out from the embryonic spinal cord (sc) of a tadpole in which HPSE2 has been experimentally downregulated. This models the somatic motor neuropathy in UFS, although only the trunk, rather than facial muscles, were studied by Roberts *et al.* [53].

We reasoned that HPSE2 deficiency causes neurological disease in UFS because, as explained earlier, most *HPSE2* mutations in UFS are predicted to be functionally null. To begin to explore this idea, we studied embryonic *Xenopus tropicalis* frogs, a vertebrate model in which gene function can be easily manipulated using morpholinos, small molecules designed to perturb RNA splicing and/or RNA translation, with the result that expression of specific proteins can be markedly downregulated. Within the normal embryonic spinal cord, cell bodies of putative neurons supplying forming skeletal muscles contained Hpse2, the frog protein highly homologous to human HPSE2 [53]. Furthermore, ablation of Hpse2 using morpholino technology caused skeletal muscle paralysis, manifest by absent hatching and escape reflexes. Motor nerves were present but, upon exiting the truncal spinal cord, they had more circuitous paths and less compactly bundled axons than controls [53], events summarized in Figure 3.

This interventional study was the first to demonstrate an *in vivo* role for HPSE2, supporting not only the contention that congenital motor neuropathy underlies UFS's clinical phenotype, but also that HPSE2 is somehow required for functional differentiation of motor nerves. As HPSE2 inhibits HPSE1's HEA, perhaps HEA overactivity causes the nerve defects by deregulating growth factor signalling (Figure 2, middle panel). While HEA overactivity has yet to be proven in this model, *Xenopus* embryos experimentally depleted of Hpse2 contain increased pERK, consistent with aberrant growth factor–mediated RTK activation. Moreover, in healthy embryo spinal cords, pERK is detected in zones where motor neuron cell bodies reside [53] (Figure 3B). Developing skeletal muscles in *Xenopus* also contain Hpse2 protein and so may play a role in the phenotype when Hpse2 is experimentally ablated. However, cells in this compartment, unlike those in the spinal cord, rarely contain pERK. The possible effects of downregulating the frog homologue of human LRIG2 has yet to be reported but, in healthy embryos, LRIG2 protein is, like HPSE2, detected in the spinal cord and developing skeletal muscles [53].

## MODELLING UFS BLADDER DYSFUNCTION IN MICE

Developing frogs do not contain a discrete functional bladder, but instead have a cloaca, acting as a simple common conduit for the gut contents and embryonic urine. Therefore, to generate an animal model of UFS's bladder disease, mice would be more appropriate. Thus far, targetted *Hpse2* null mutant mice (i.e. with the gene precisely excised using Cre/LoxP technology) have not been described, but two groups have reported their initial studies of ‘gene trap’ mutants. In this model, a retroviral insertion into the *Hpse2* gene produces a truncated transcript; if any HPSE2 protein were to be generated, it would be unlikely to have normal function. Stuart *et al.* [27] reported autopsies of homozygous *Hpse2* mutant mice in the first month of their postnatal lives. Their bladders contained urine significantly more often than wild-type or heterozygous littermates; moreover, there was no evidence of anatomical obstruction of the urethra, so the phenotype resembled functional BOO found in humans with UFS. Subsequently Guo *et al.* [54] reported high hydrostatic pressures within incompletely emptying bladders of homozygous *Hpse2* gene trap mice [54]. They also noted that these mice displayed more frequent voiding than wild-types and that the volumes of urine voided was less than normal [54], as occurs in UFS.

Stuart *et al.* [27] measured similar levels of epithelial (*uroplakin 3A*) and smooth muscle (*α-smooth muscle actin* and *myosin heavy chain 11*) transcripts in homozygous and wild-type littermate bladders harvested at 1 and 14 days after birth. These results argue against a primary epithelial or myogenic defect and indirectly support a neurogenic pathogenesis. Guo *et al.* [54] found that *Hpse2* mutant bladders were fibrotic, with biochemical evidence of increased transforming growth factor β signalling activity. These changes, however, may be secondary to increased stretch and pressures, themselves caused by functional BOO. Indeed, BOO caused by experimental urethral ligation causes striking secondary changes in the cell and molecular biology of developing bladders [55]. Further work is now needed on mouse *Hpse2* mutant bladders, with a focus on the function and fine structure of nerves within the bladder, their associated ganglia and connections with the spinal cord.

## ENVISIONING NOVEL THERAPIES

Of interest to nephrologists, mice that have been genetically engineered to overexpress HPSE1 have proteinuria [56]. Urine and plasma HPSE1 levels are elevated in people with diabetes mellitus, correlating with hyperglycaemia [57]. In mice with experimental diabetes, HPSE1 is upregulated in glomeruli in association with HS depletion in glomerular basement membranes [46]. Mice treated with the HEA inhibitor SST0001, as well as mice with genetic deletion of *Hpse1*, are protected from developing diabetic nephropathy [46]. An explanation for these observations is that high glucose levels upregulate HPSE1 and that the subsequent HEA-mediated loss of negatively charged HSPGs perturbs the macromolecular barrier function of the glomerular basement membrane, with subsequent proteinuria.

If a main role of HPSE2 is to antagonize HEA, and individuals with *HPSE2* mutations lack functional HPSE2, then UFS can be seen as an ‘HEA overactive’ disease, at least in relation to the nervous system. So, by analogy with diabetic nephropathy, chemicals such as SST0001 and other heparin-like compounds that inhibit HEA [58] may serve as novel treatments for UFS. This hypothesis could be tested in available frog and mouse animal models. Of note, HEA inhibitors have entered clinical trials in cancer [59–61].

Experimental thoracic spinal cord transection in rats leads to frequent bladder contractions with high intravesical pressures, a phenotype resembling UFS [62]. pERK was upregulated in the lumbar region of these animals' spinal cords and when they were administered PD98059, a specific inhibitor of ERK phosphorylation, bladder dysfunction was ameliorated [62]. Moreover, when rat bladders were experimentally inflammed by cyclophosphamide, pERK upregulation was detected in the lumbar spinal cord in zones occupied by projections of bladder afferent neurons [63]. Administration of PD98059 decreased the frequency of contractions in inflamed bladders [63]. These biochemical observations are notable because *Xenopus* embryos experimentally depleted of Hpse2 have upregulated pERK [53]. Perhaps chemical blockade of pERK would ameliorate bladder dysfunction in UFS. As for HEA inhibitors, chemicals that manipulate intracellular signalling pathways involving ERK are being explored as treatments for cancers [64].

Despite these ideas, however, the potential difficulties of targeting biological therapies to developing organs *in vivo* should not be underestimated. Although there are no existing examples that target bladder nerves, the following observations are encouraging. First, Picconi *et al.* [65] showed that intravenous administration of an adeno-associated virus subtype to pregnant mice led to transplacental passage of the virus and transduction of a reporter gene into a wide variety of foetal organs, including the renal tract. The same study showed that kidneys could be specifically targetted by driving the reporter gene from a glomerulus-specific promoter [65]. Second, there are similarities between peripheral nerve disease in UFS and another congenital neuropathy called spinal muscular atrophy. As reviewed by Faravelli *et al.* [66], following successful proof of principle animal experiments, the US Food and Drug Administration has approved a Phase I clinical trial (NCT02122952) in which intravenously administered adenovirus will be used to deliver the defective gene to affected spinal muscular atrophy individuals after birth.

## WIDER IMPLICATIONS OF UFS MOLECULES

Might lessons learned about UFS have relevance for other diseases? We have already alluded to biochemical mechanistic analogies between diabetic nephropathy and UFS. UFS is a discrete clinical disorder, but its urinary tract abnormalities, including bladder dyssynergia and VUR, overlap with features of Hinman–Allen syndrome (or ‘non-neurogenic neurogenic bladder’) [67], itself at the severe end of a spectrum of LUT disorders including primary VUR, which affects 1% of infants and is often familial [68]. In fact, despite analyses [27], *HPSE2* mutations have not yet been directly implicated in causing these overlapping disorders. LRIG2 mutations have yet to be sought in familial primary VUR. However, the report of an individual [26] who carries biallelic *LRIG2* mutations and is affected by non-neurogenic neurogenic bladder and ESRD, but lacking UFS facial features, suggests wider implications for UFS genes.

## CONFLICT OF INTEREST STATEMENT

The authors have no conflicts of interest to declare. The figures are originals and not reproduced from other publications.
